# Perceptual learning improves spatial contrast sensitivity in older adults

**DOI:** 10.3389/fnins.2025.1681856

**Published:** 2025-12-17

**Authors:** Yong Tang, Ju Liang, Yifeng Zhou

**Affiliations:** 1 Hefei National Laboratory for Physical Science at the Microscale, Division of Life Sciences and Medicine, University of Science and Technology of China, Hefei, China; 2 Department of Medical Technology, Anhui Institute of Medicine, Hefei, China; 3 Anhui Provincial Center for Maternal and Child Health Genetics, Hefei, China; 4 Sichuan Fine Arts Institute, Chongqing, China; 5 State Key Laboratory of Brain and Cognitive Science, Institute of Psychology, Chinese Academy of Science, Hefei, China

**Keywords:** aging, contrast perceptual learning, spatial contrast sensitivity, visual acuity, visual plasticity

## Abstract

**Introduction:**

Widespread visual deficits accompany normal aging, with most attributed to functional degradation of the visual cortex. Although perceptual learning can improve many visual functions in older adults, it remains unclear whether it can enhance spatial contrast sensitivity, a fundamental visual function known to decline significantly from around age forty.

**Methods:**

To address this, we trained 29 older adults and 18 young controls using contrast perceptual learning. Training was conducted at seven spatial frequencies (from low to high) and/or at the individual cut-off spatial frequency. Spatial contrast sensitivity function (SCSF) and visual acuity (VA) were measured before and after training.

**Results:**

Training induced substantial improvements in both SCSF and VA in older adults, which were retained for at least several months. Analysis of transfer effects revealed that, compared to young controls, older adults exhibited a characteristic low-frequency shift in peak improvement and a slightly broader bandwidth.

**Discussion:**

These results may be associated with age-related alterations in neuronal response properties within the primary visual cortex. Our findings demonstrate substantial neural plasticity in the aging visual system and support the potential of perceptual learning as a clinically viable intervention for mitigating age-related visual decline.

## 1 Introduction

A great number of visual functions decline during normal aging (Spear, 1993; Owsley, 2010), which has been suggested to be associated with increased risk of falls, injuries from falls, mortality and decreased health-related quality of life (Harwood, 2001; Salonen and Kivela, 2012). With the increasing aging population worldwide, it has attracted growing attention.

Many studies have suggested that the declines in visual function found in older adults cannot be solely accounted for by senescent optical changes (Owsley et al., 1983; Ball and Sekuler, 1986; Betts et al., 2007; Tang and Zhou, 2009). And subcortical neural changes due to normal aging are relatively minor (Spear et al., 1994; Kim et al., 1996; Harman et al., 2003; Feng et al., 2007). These findings naturally suggest a cortical origin for these functional declines. Indeed, electrophysiological studies have indicated that many neuronal response properties, such as latency (Wang et al., 2005), orientation and direction selectivity (Schmolesky et al., 2000; Leventhal et al., 2003; Yu et al., 2006; Liang et al., 2010; Fu et al., 2012), spatial and temporal frequency tuning properties (Yuan et al., 2014), contrast sensitivity (the half saturation contrast in neuronal contrast response functions) (Yang et al., 2008) and motion speed selectivity (Yang et al., 2009), are significantly degraded in old monkeys. Similar results have also been found in old cats (Hua et al., 2006) and rats (Wang et al., 2006). All these findings support the hypothesis that most of the declines in visual function during normal aging can be attributed to the functional degradation of the visual cortex.

On the other hand, some evidence has suggested that visual perceptual learning, repetitive training with a specific visual perceptual task, could refines the neuronal response properties in some cortical regions, such as V1 (Schoups et al., 2001; Hua et al., 2010; Ren et al., 2016) and V4 (Adab and Vogels, 2011). These neurophysiological changes may constitute the neural basis for the improved behavioral performance observed after perceptual learning (Gilbert, 1994; Fahle, 2004; Lu et al., 2011). For example, Hua and his colleagues examined the effects of training in grating orientation identification on both perceptual (behavior) and neuronal (derived from the contrast sensitivity of the individual neurons) contrast sensitivity functions of cats using combined psychophysical measurements with extracellular single-unit recording (Hua et al., 2010). They found that trained cats showed significantly higher perceptual and neuronal contrast sensitivity in V1 when compared to untrained cats. Additionally, the neuronal contrast sensitivity functions derived from the contrast sensitivity of individual neurons were highly correlated with behaviorally determined perceptual contrast sensitivity functions in both trained and untrained cats. Using a coarse orientation discrimination task, Adab and Vogels also found substantial increases in the sensitivity of V4 neurons in monkeys during the course of perceptual learning (Adab and Vogels, 2011). These improvements in neural discrimination were comparable to behavioral improvements, and were related to decreased response variability and an increase of the difference between the mean responses for the two trained orientations.

In view of this, visual perceptual learning may be a good approach for visual recovery of normal older adults. Indeed, this assumption has been supported by some studies. For example, after 1,152 training trials, older adults demonstrated significant improvement in texture discrimination, reaching a performance level comparable to that of young adults (Andersen et al., 2010). Motion perception of older adults, both in fine (Ball and Sekuler, 1986) and coarse (Bower and Andersen, 2012; Bower et al., 2013) direction discrimination tasks, was significantly improved after several hundreds to thousands training trials. Following several days of training with stimuli embedded in multiple external noise levels, older adults showed significant improvements in orientation discrimination and near visual acuity (Deloss et al., 2014, 2015). Similar results were also found for some other visual functions, such as letter and brightness discrimination (Ratcliff et al., 2006), global visual form perception (Mayhew and Kourtzi, 2013), contour integration (McKendrick and Battista, 2013) and visual search (Rogers and Fisk, 1991). However, whether the spatial contrast sensitivity function (SCSF), a basic descriptor of visual function which has been shown to be degraded significantly as early as about forty years old (Owsley et al., 1983), can be improved in older adults through perceptual learning is still unknown. Note that although the previous studies have shown that the contrast sensitivity of older adults can be improved at a relatively low spatial frequency (1.5 c/d) through an orientation discrimination task, less is known whether the same improvement occurs at high spatial frequencies. We therefore investigated this issue in this study.

We initially trained older adults in Group 1 using a contrast detection task with sine-wave gratings presented at each individual’s cut-off spatial frequency — a method previously demonstrated to aid visual recovery of the spatial contrast sensitivity function in amblyopia (Zhou et al., 2006). Post-training assessments revealed substantial improvements not only at the trained cut-off, i.e., high, frequency but also at adjacent spatial frequencies, collectively elevating the SCSF. However, when we analyzed the transfer effects of perceptual learning using the same method as that used in a previous study on amblyopia (Huang et al., 2008), improvements were found to be more pronounced at slightly lower spatial frequencies than at the trained frequencies. This finding suggested that perceptual learning in older adults might be more readily induced at lower spatial frequencies, yielding stronger training effects than at the cut-off frequency. To test this hypothesis, we designed a two-stage training protocol for Group 2: the first stage involved prolonged but low-intensity training across a range of spatial frequencies (from low to high), while the second stage focused exclusively on cut-off spatial frequency training. The first stage of training was informed by our prior finding that mixed spatial frequency training improves SCSF in amblyopia (Zhou et al., 2006). The objective of the second stage was twofold: to test the aforementioned hypothesis and to evaluate whether the initial training stage was sufficient to induce maximal effects in older adults after performance had reached a clear plateau. Results showed that both stages of training induced substantial improvements. Moreover, Group 2 exhibited greater overall SCSF improvements than Group 1. This superior outcome was primarily attributable to gains at intermediate spatial frequencies, most of which were achieved during the first training stage. Transfer effect analyses of the second-stage training in Group 2 again revealed a clear shift in peak improvement toward lower spatial frequencies, demonstrating a distinctive improvement pattern in older adults. Finally, to compare contrast perceptual learning characteristics between younger and older adults, Group 3 (young adults) underwent training using the same protocol as Group 1 and the second stage of Group 2.

## 2 Materials and methods

### 2.1 Subjects

A total of 29 older adults and 18 normal young controls participated in this experiment. Older adults were randomly assigned into group 1 (7 males and 8 females, age range: 64–73 years, average age: 67.1 ± 2.6 years) and group 2 (7 males and 7 females, age range: 65–76 years, average age: 68.2 ± 3.6 years), while all young controls (9 males and 9 females, age range: 23–26 years, average age: 23.6 ± 0.2 years) were in group 3.

All subjects had normal or corrected-to-normal visual acuity (better than 20/25) and were free from ocular diseases. The Mini-Mental State Examination (MMSE) was performed on these subjects to exclude probable dementia. Alcoholism, stroke and depression were also exclusion criteria. All subjects were naive to the purpose of the psychophysical experiments. This research has been approved by the ethics committee of the University of Science and Technology of China, and was performed in accordance with the ethical standards laid down in the 1964 Declaration of Helsinki. The written informed consent was obtained from all participants before participation.

### 2.2 Apparatus and visual stimuli

The stimuli were generated in real time using Matlab programs based on Psychtoolbox version 3.0, and were displayed on a gamma-corrected 17-inch Sony G220 CRT monitor with a spatial resolution of 1600*1200 pixels, a refresh rate of 75 Hz, and a mean luminance of 37 cd/m^2^. A special circuit (Li et al., 2003) was used to produce 14 bits of gray levels. All stimuli were presented in the center of the screen, and were viewed biocularly at a distance of 2.28 m in a dimly lit room.

The stimuli were vertical sine-wave gratings (Equation 1). The luminance profile at point (x, y) of the stimuli is defined as:


$$l(x,y)=L_{\text{mean}}\{1+C•\sin\{2\mathrm{\pi f}(y\cos\theta -x\sin\theta )+\phi \}\}$$ (1)

where $L_{\text{mean}}$ is the background luminance of the display; C is the grating contrast; f is the spatial frequency of the sine wave grating; θ represents the orientation of the grating (here set to 90); and ϕ is the (random) initial spatial phase.

The stimuli subtended 3.6° × 3.6° of visual angles and were presented in the center of the screen. To minimize edge effects, a 0.5 deg. half-Gaussian ramp was added to each side of the stimulus to blend the stimuli to the background.

### 2.3 Experimental design and procedure

The experiment consisted of three consecutive phases: pre-training assessment, training, and post-training reassessment. In pre- and post-training assessments, spatial contrast sensitivity function (SCSF) and visual acuity were measured. The measurement of SCSF contained seven blocks with 100 trials per block. Different spatial frequencies (1, 2, 4, 8, 16, 24 and 32 c/d) were randomly intermixed in each block. Visual acuity was assessed with the Chinese Tumbling E Chart and defined as the score associated with 75% correct judgments.

The three groups used somewhat different training protocols, as detailed in Table 1.

**Table 1:** Training parameters used in each experimental group.

Parameters	Group 1	Group 2, Stage 1	Group 2, Stage 2	Group 3
Age (years)	67.1 ± 2.6	68.2 ± 3.6	68.2 ± 3.6	23.6 ± 0.2
Number	15	14	14	18
Number of training sfs	1	7	1	1
Training sf (cycles/degree)	Cut-off sf mean = 29.0 SE = 0.5	Seven sfs (1, 2, 4, 8, 16, 24 & 32)	Cut-off sf mean = 27.2 SE = 1.1	Cut-off sf mean = 30.6 SE = 0.7
Total trials in a training session	1,000	1,000	1,000	1,000
Trials per training sf	1,000	~143	1,000	1,000
Total number of training sessions	10–14 mean = 11.6 SE = 0.3	14–16 mean = 14.6 SE = 0.2	10–14 mean = 11.1 SE = 0.3	10–12 mean = 10.4 SE = 0.2

All Subjects in group 1 and most of the subjects in group 3 received training with sine-wave gratings at each individual’s cut-off spatial frequency, defined as the spatial frequency at which the estimated contrast threshold from pre-training SCSF measurements was 0.5. For some subjects in group 3, whose cut-off spatial frequencies were larger than 32 c/d, the training spatial frequencies were set to 32 c/d. The training spatial frequencies were 29.0 ± 0.5 c/d (range: 24–32 c/d) in group 1, and were 30.6 ± 0.7 c/d (range 25–32 c/d) in group 3.

Training of group 2 comprised two stages. In the first training stage, subjects practiced the SCSF task over the entire range of spatial frequencies tested in the SCSF measurement, i.e., discrete training at all spatial frequencies. And in the second training stage, subjects received the same training protocol as group 1 and 3, with their cut-off spatial frequencies ranging from 24 to 32 c/d (average value: 27.2 ± 1.1 c/d).

An average of 11.6 (range: 10–14), 14.6 (range: 14–16), 11.1 (range: 10–14), and 10.4 (range: 10–12) training sessions were performed in group 1, the first stage and the second stage of group 2, and group 3, respectively. In most cases, training was terminated after first three consecutive sessions with similar performance. While for the first training stage of group 2, training phase was somewhat prolonged, and was terminated after five consecutive sessions with similar performance.

Each training session contained 1,000 trials and only one session was run per day. Note that for group 1, 3 and the second training stage of group 2, this setting provided high training intensity at the training spatial frequency (1,000 trials per day). While for the first training stage of group 2, which covered a wider range of the training spatial frequency, this setting provided low training intensity at one training spatial frequency (about 143 trials per day), which may result in less training effects, especially at high spatial frequencies, in older adults even after their training phase had been prolonged and had already reached the plateau.

In most cases, the contrast thresholds (converging at 79.4% correct) were measured for all subjects by using a temporal two-alternative-forced-choice design and a two-down one-up staircase procedure (Levitt, 1971). Contrast sensitivity (reciprocal of contrast threshold) was used for data analysis.

All trials were initiated by the subjects. In each trial, the stimulus was presented for 300 ms. Then the subject indicated her/his decision with a keyboard button press. Audio feedback was provided in training sessions but not in the SCSF measurement. Before the formal experiment began, all participants were given a short session of 30–50 trials to familiarize them with the task.

### 2.4 Statistical analysis and model fit

Pre- and post-training visual acuity and contrast sensitivity at the training spatial frequency were compared using within-subject *t*-test. SCSFs in the beginning and the end of training were compared using within-subject ANOVA.

The magnitude of improvement for each measure (contrast sensitivity and visual acuity) was calculated as:


Impindividual=20log10Measurepost−trainingMeasurepre−trainingdb (2)

In the training stage, the magnitude of improvement was calculated as:


$$I_{\text{session}(i)}=20\log_{10}\frac{\text{Measur}e_{\text{session}(i)}}{\text{Measur}e_{\text{session}(1)}}\mathrm{db}$$ (3)

When calculating the bandwidth of perceptual learning, only subjects who showed statistically significant improvements at the training spatial frequency were included (the slope of the learning curve is at least marginally different from zero, *p* < 0.10). Based on this criterion, S20, S26 and S27 from Group 2, and S30, S35, S36, S40, S42, S43, S45 and S47 from Group 3 were excluded.

The bandwidth of contrast perceptual learning at the cut-off spatial frequency was estimated with the following procedure: (1) The difference between the pre- and post-training SCSF for each observer was calculated; (2) For each subject, the magnitudes of contrast sensitivity improvements were normalized to that at the training spatial frequency; spatial frequencies were normalized to the training frequency; (3) Normalized spatial frequencies ( $\log_{2}\frac{\mathrm{sf}}{sf_{\text{training}}}$ ) were then divided into eight bins: [5, 4), [4, 3), [3, 2), [2, 1), [1, 0.5), [0.5, 0), 0, and (0, 0.5]. Data within each bin were averaged, weighted by the reciprocals of their standard deviations; (4) The normalized contrast sensitivity improvements were fit with the following Gaussian functions:


$$\log_{10}\frac{CS_{\text{post}-\text{training}}(\mathrm{sf})}{CS_{\mathrm{pre}-\text{training}}(\mathrm{sf})}=\mathrm{Am}p^{*}\exp(-1^{*}{\frac{\left(\log_{2}\frac{\mathrm{sf}}{sf_{\text{training}}}\right)}{\sigma^{2}}}^{2})$$ (4)

$$\log_{10}\frac{CS_{\text{post}-\text{training}}(\mathrm{sf})}{CS_{\mathrm{pre}-\text{training}}(\mathrm{sf})}=\mathrm{Am}p^{*}\exp(-1^{*}{\frac{\left(\log_{2}\frac{\mathrm{sf}}{sf_{\text{training}}}\right)}{\sigma^{2}}}^{2})+\text{baseline}$$ (5)

$$\log_{10}\frac{CS_{\text{post}-\text{training}}(\mathrm{sf})}{CS_{\mathrm{pre}-\text{training}}(\mathrm{sf})}=\mathrm{Am}p^{*}\exp(-1^{*}{\frac{\left(\log_{2}\frac{\mathrm{sf}}{sf_{\text{training}}}-\text{peak}\right)}{\sigma^{2}}}^{2})$$ (6)

$$\log_{10}\frac{CS_{\text{post}-\text{training}}(\mathrm{sf})}{CS_{\mathrm{pre}-\text{training}}(\mathrm{sf})}=\mathrm{Am}p^{*}\exp(-1^{*}{\frac{\left(\log_{2}\frac{\mathrm{sf}}{sf_{\text{training}}}-\text{peak}\right)}{\sigma^{2}}}^{2})+\text{baseline}$$ (7)

where Amp , peak , σ and baseline are the amplitude, the peak location, the standard deviation and the baseline of the Gaussian function.

The model fitting procedures were implemented in Matlab with the Curve Fitting Toolbox (Mathworks). The sum of squared differences ( ∑(Imptheory(sf)−Impmeasured(sf))2 ) between the measured improvement ( Impmeasured(sf) ) and the model-predicted improvement ( Imptheory(sf) ) was minimized. The goodness-of-fit was evaluated by the $r^{2}$ statistic:


r2=1.0−∑(Imptheory(sf)−Impmeasured(sf))2∑[Impmeasured(sf)−mean(Impmeasured(sf))]2 (8)

Because the models (Equations 1–4) described above are nested (i.e., their parameters are proper subsets or supersets of one another), an *F*-test could be used to compare a full to a reduced model (Hays, 1988):


$$F(df_{1},df_{2})=\frac{(r_{\text{full}}^{2}-r_{\text{reduced}}^{2})/df_{1}}{(1-r_{\text{full}}^{2})/df_{2}}$$ (9)

where $df_{1}=k_{\text{full}}-k_{\text{reduced}}$ and $df_{2}=N-k_{\text{full}}-1$ . The k ’s are the number of parameters in each model and N is the number of predicted data points. The model which had the fewest parameters but provided a fit that was statistically equivalent to the other models, was selected as the best model.

The spatial transfer bandwidth of perceptual learning was defined as:


$$B=2\sqrt{\ln(2)}*\sigma$$ (10)

where σ is the spread of the Gaussian function.

The standard error of the mean (SE) of each model parameter for the best-fitting model was estimated using a re-sampling method (Maloney, 1990). Each data point was assumed to have a Gaussian distribution with its mean value and standard deviation equal to the estimated values from the experimental data. Then a set of data points could be generated by sampling each of the Gaussian distributions once. We repeated this process to generate 1,000 datasets, each of which was used to find a curve-fit; the SDs of these 1,000 parameter sets provided error estimates for the model parameters.

## 3 Results

### 3.1 Learning curves

**Figure 1:**
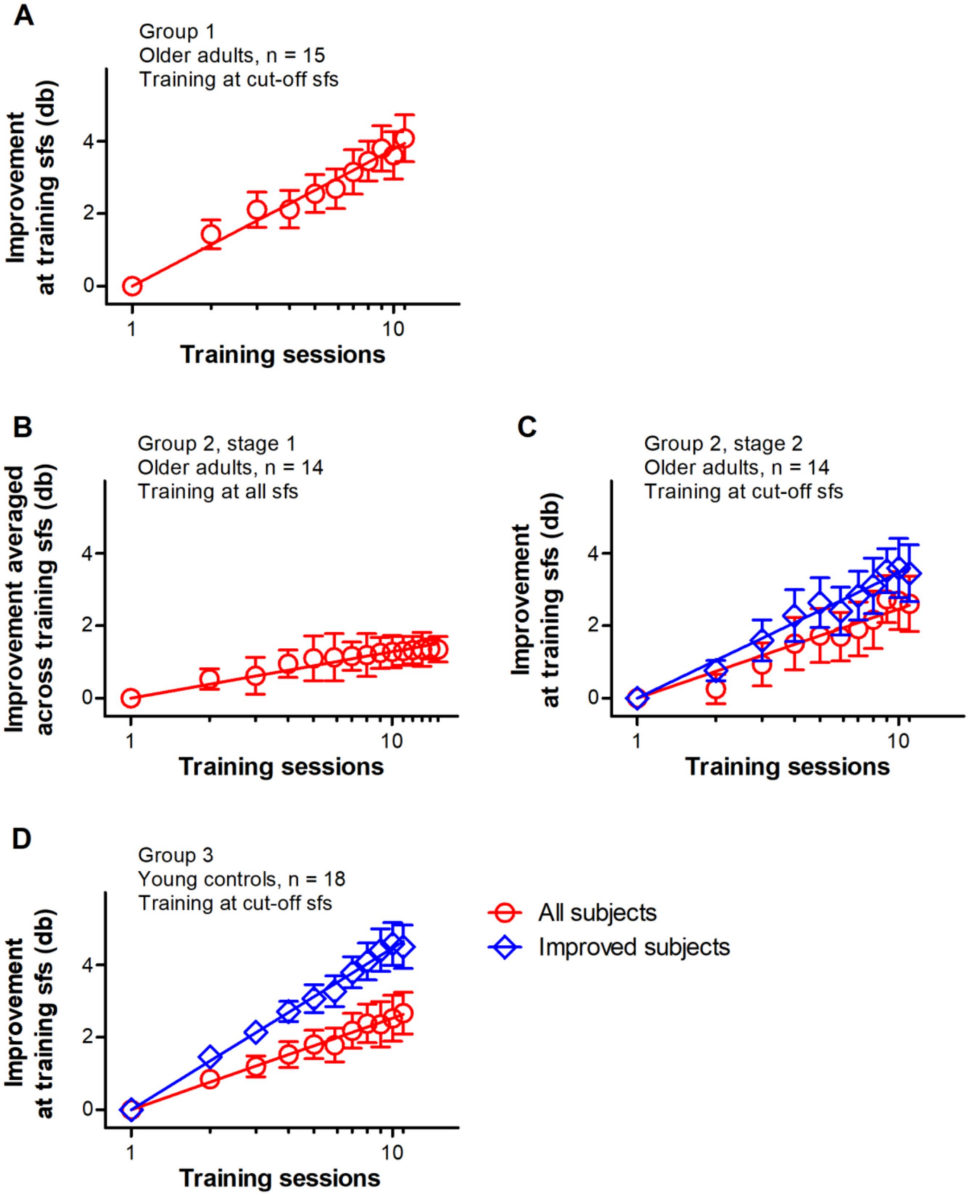
Average learning curves for three groups. The number of training sessions varied between subjects. And only the first ten **(A,C,D)** or fourteen **(B)** training sessions are illustrated here. As a result, when taking account of all training sessions and the post-training evaluation, the improvement is slightly larger than what is showed here. Note that the first session represents the pre-training SCSF measurement, and its improvement value is set to 0. The red symbols and lines represent the average of all subjects, and the blue represents those who exhibited significant learning during training. Data were fitted with linear functions. Error bars indicate between-subject SE.

Average learning curves for the three groups are shown in Figure 1, and all groups demonstrated statistically significant improvements.

For Group 1, training at the cut-off spatial frequency significantly improved the contrast sensitivity of older adults (*p* < 0.001). When only considering the data of the pre-training measurement and the first ten training sessions, training improved contrast sensitivity with an average of 3.79 db per log unit of training session (*p* < 0.001), with the average learning curve is shown in Figure 1A.

For Group 2, all subjects received two stages of training. The learning curves for these two stages are shown in Figures 1B,C, respectively.

In the first stage of training, repeated SCSF measurements (i.e., repeated exposure to stimuli with a large range, from low to high, of spatial frequencies) induced substantial improvements of contrast sensitivity averaged across spatial frequencies (*p* < 0.001). When only considering the data of the pre-training measurement and the first fourteen training sessions, the average learning curve showed a slope of 1.28 db per log unit of training session, which can be seen in Figure 1B. Note that the last 3 data points in the learning curve are very similar to each other, demonstrating that a plateau has already been reached in this stage.

In the second stage, contrast sensitivity at the cut-off spatial frequency significantly improved after training (*p* = 0.001). As a group, training improved contrast sensitivity with an average of 2.49 db per log unit of training session, which can be seen in the learning curve (Figure 1C). When excluding the data of three subjects (S20, S26 and S27) who did not show significant improvements during training, the slope of learning curve is 3.46 db per log unit of training session.

**Figure 2:**
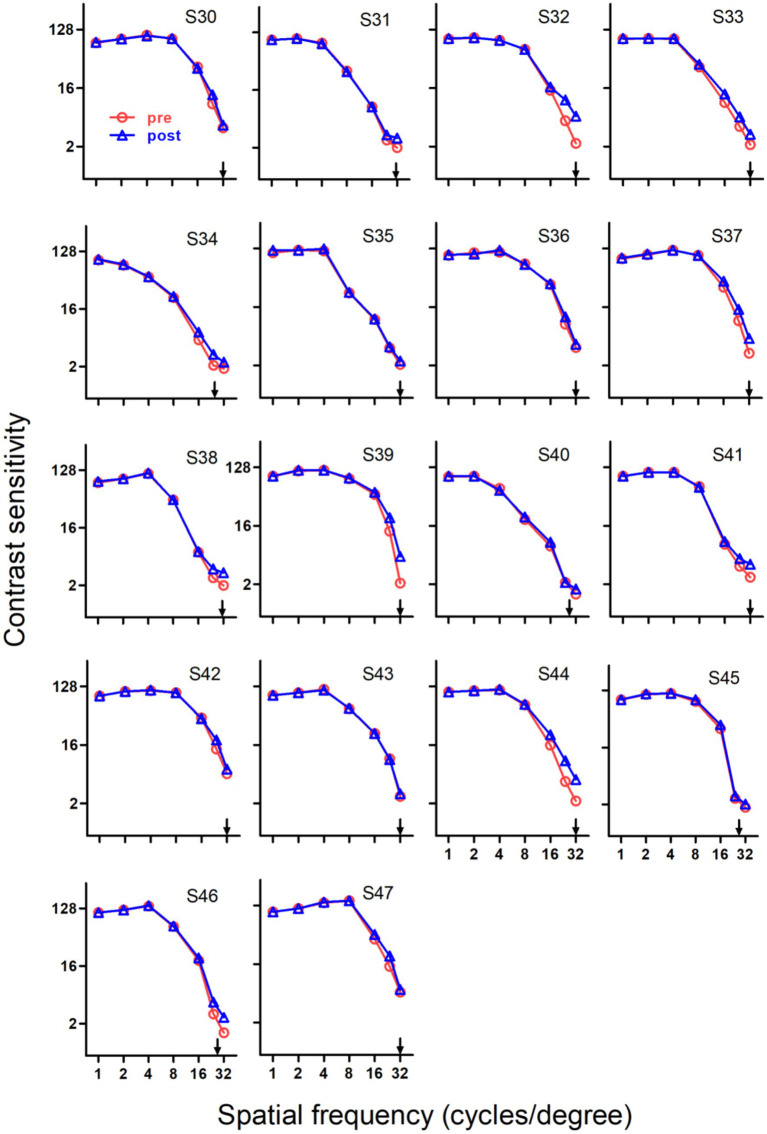
Pre- and post-training spatial contrast sensitivity functions of normal young adults in Group 3. After training, ten subjects (S31, S32, S33, S34, S37, S38, S39, S41, S44 and S46) showed significant improvements in spatial contrast sensitivity function, while other eight subjects (S30, S35, S36, S40, S42, S43, S45 and S47) only exhibited minimal improvement. Red circles, pre-training; blue triangles, post-training; arrows, training frequency; error bars, within-subject SE.

For the control group (Group 3), training at the cut-off spatial frequency significantly improved the contrast sensitivity (*p* < 0.001). The average learning curve is shown in Figure 1D. As a group, training improved contrast sensitivity with an average of 2.53 db per log unit of training session. Note that 8 subjects (S30, S35, S36, S40, S42, S43, S45 and S47) in Group 3 did not show significant improvements during training, consistent with the results of SCSF measurements pre- and post-training (Figure 2). When excluding data of these subjects, the slope of learning curve is 4.47 db per log unit of training session, and the improvements between the pre- and post-training measurements were 4.97 db (SE = 0.65 db) averaged across subjects.

### 3.2 Improvements in SCSF

**Figure 3:**
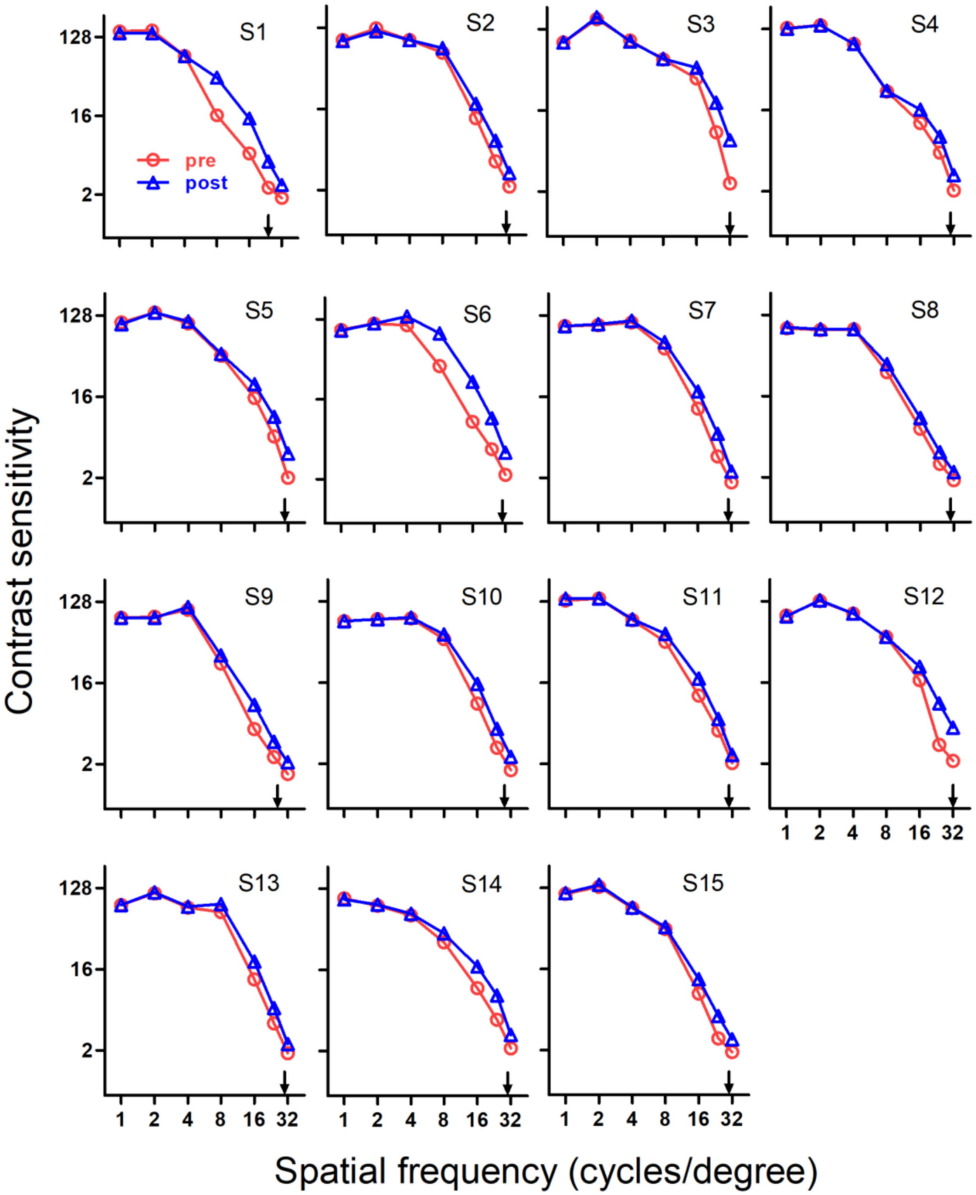
Pre- and post-training spatial contrast sensitivity functions of older adults in Group 1. All subjects exhibited significant improvements in spatial contrast sensitivity function after training. Red circles, pre-training; blue triangles, post-training; arrows, training frequency; error bars, within-subject SE.

**Figure 4:**
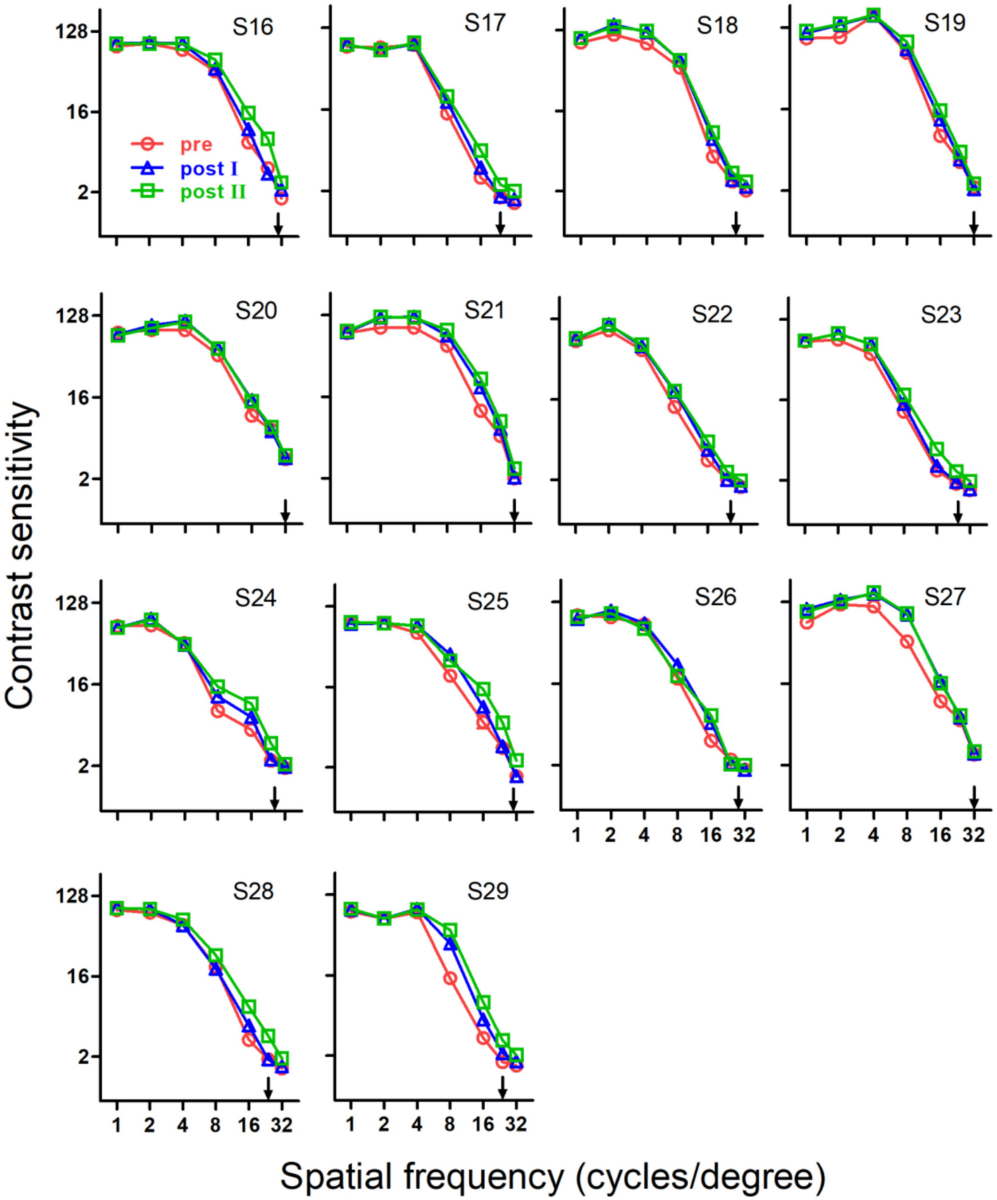
Pre- and post-training spatial contrast sensitivity functions of older adults in Group 2. After two stages of training, all subjects exhibited significant improvements in spatial contrast sensitivity function. Note that at the second training stage, three subjects (S20, S26 and S27) demonstrated virtually no improvement. Red circles, pre-training; blue triangles, post-training of stage 1; green squares, post-training of stage 2; arrows, training frequency; error bars, within-subject SE.

Training induced substantial improvements in SCSF for three groups, which can be seen in Figures 2–4, respectively.

**Figure 5:**
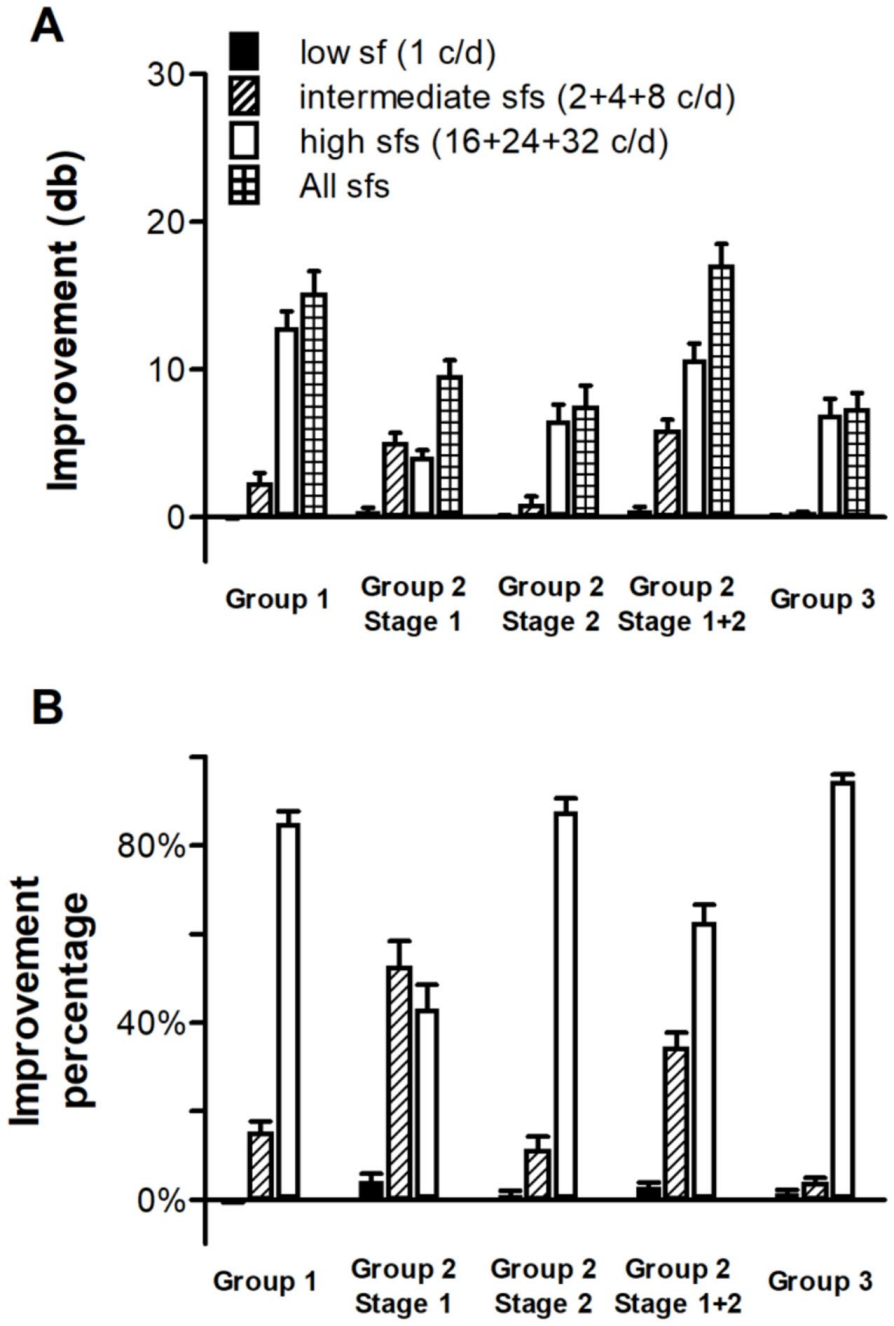
Comparisons of improvements **(A)** and the improvement percentages **(B)** at low, intermediate and high spatial frequencies in three groups. The improvement patterns of older adults (Groups 1 and 2) differed from those of young controls (Group 3), showing more pronounced gains at intermediate spatial frequencies. Error bars indicate between-subject SE.

For Group 1, substantial improvements in contrast sensitivity were not only found at the training site, but also found at adjacent spatial frequencies, which can be seen in Figures 3, 5A. This led to significant changes of the whole SCSF (*p* < 0.001). The sum of improvements at low (1 c/d), intermediate (2, 4 and 8 c/d) and high (16, 24 and 32 c/d) spatial frequencies were −0.05 ± 0.05, 2.30 ± 0.70 and 12.86 ± 1.10 db, respectively. Note that these improvements were strongly dependent on spatial frequency (*p* < 0.001), with the majority of the improvements occurring at high spatial frequencies.

In the first training stage of Group 2, the repeated exposure to stimuli with a large range of spatial frequencies induced substantial improvements at almost all spatial frequencies. As shown in Figures 4, 5A, the greatest improvements occurred at intermediate spatial frequencies. The sum of training-induced improvements at low (1.0 c/d), intermediate (2.0,4,0 and 8.0 c/d) and high spatial frequencies (16.0, 24.0 and 32.0 c/d) were 0.41 ± 0.24, 5.01 ± 0.69 and 4.11 ± 0.42 db, respectively.

In the second stage of Group 2, training effects transferred to adjacent spatial frequencies, similar to those in Group 1. As a result, the whole SCSF exhibited statistically significant improvements (*p* < 0.001). It is clear from Figures 4, 5A that most of the improvements occurred at high spatial frequencies, similar to those observed in Group 1. The sum of training-induced improvements at low, intermediate and high spatial frequencies were 0.08 ± 0.07, 0.84 ± 0.55 and 6.57 ± 1.05 db, respectively.

Note that the majority (about 83%) of the improvements in this stage occur at high spatial frequencies. Given the fact that training at high spatial frequencies but with low training intensity (for more details, see Table 1) had already conducted in the first stage, and the prolonged training of the first stage had reached a plateau, the significant improvements, mainly at high spatial frequencies, of contrast sensitivity in the second stage might suggest that the effect of contrast perceptual learning in the first stage, at least at the cut-off spatial frequency, was much restricted by the reduced training intensity, i.e., the training-induced improvements at the cut-off spatial frequency strongly depended on the training intensity.

The pre- and post-training SCSF results for Group 3 are presented in Figures 2, 5A. It is clear that the majority (10 out of 18) of young controls exhibited substantial improvements in SCSF, while the remaining eight subjects (S30, S35, S36, S40, S42, S43, S45, and S47) in Group 3 did not. We note that some young controls exhibited no improvement after training, which replicates a finding from a previous study (Huang et al., 2008) using the same training protocol. As a group, the subjects exhibited a statistically significant upward shift in the whole SCSF after training (*p* < 0.001). The improvements at low, intermediate and high spatial frequencies were 0.11 ± 0.04, 0.28 ± 0.10 and 6.92 ± 1.10 db, respectively. These data demonstrate that almost all of the improvement occurred at high spatial frequencies.

### 3.3 Comparisons of improvements in SCSF

Comparisons of improvements in three groups are shown in Figure 5.

The magnitude of improvements was similar between Group 1 and Group 2. The total improvements in the first (9.53 ± 1.09 db) and second stage (7.49 ± 1.42 db) of Group 2 were both less than those in Group 1 (15.11 ± 1.54 db), as shown in Figure 5A. But taking two stages together, Group 2 exhibited slightly greater improvements, although this difference was not significant in statistics (*p* = 0.381). These data may suggest that the cutoff training regimen for Group 1 is more efficient, since Group 1 achieved comparable improvements to Group 2 with fewer training sessions and stages.

However, the improvement structure and pattern were somewhat different between these two groups. As shown in Figure 5A, the improvements at intermediate spatial frequencies in Group 2 (5.85 ± 0.74 db), which mainly came from the first stage, were significantly greater (*p* = 0.002) than those in Group 1 (2.30 ± 0.70 db). While at high spatial frequencies, the improvements in Group 2 (10.68 ± 1.10 db), which came from both the first and the second stages, were slightly less but not statistically different (*p* = 0.174) from those in Group 1 (12.86 ± 1.10 db).

These two groups also exhibited different improvement patterns, which can be seen in Figure 5B. The improvement percentages at low, intermediate and high spatial frequencies in group 1 were −0.30, 15.21 and 85.10%, respectively. In contrast, they were 4.26, 52.58 and 43.15% in the first stage of group 2, and 1.18, 11.21 and 87.71% in the second stage. The improvement pattern of the former was significantly different from group 1 ( $\chi_{2}^{2}$ = 39.790, *p* < 0.001), whereas the latter not ( $\chi_{2}^{2}$ = 1.677, *p* = 0.434). As a whole, the improvement pattern of group 2 was also significantly different from that in group 1 ( $\chi_{2}^{2}$ = 13.638, *p* = 0.001), with a larger improvement percentages at intermediate spatial frequencies while a less percent at high spatial frequencies. Even without accounting for training intensity, these findings demonstrate that training at intermediate spatial frequencies could enhance SCSF in older adults. This beneficial effect cannot be fully replicated by training solely at the cut-off (i.e., high) spatial frequency.

As shown in Figure 5, the improvement structure and pattern were similar between Group 3 (young controls) and the second stage of Group 2 (older adults). The total improvements in young adults (7.31 ± 1.11 db) were comparable to those in the second stage of Group 2 (*p* = 0.920). Moreover, almost all improvements in young adults occurred at high spatial frequencies, similar to those found in the second stage of Group 2 ( $\chi_{2}^{2}$ = 3.863, *p* = 0.145). However, no such similarity was found with Group 1. The total improvements in young adults were significantly lower than those in Group 1 (*p* < 0.001). And improvements at intermediate spatial frequencies accounted for a higher proportion of the total gains in Group 1 than in Group 3, leading to statistically significantly different improvement patterns ( $\chi_{2}^{2}$ = 8.919, *p* = 0.012). These findings suggest that only after training at intermediate spatial frequencies could older adults exhibit perceptual learning characteristics similar to young controls.

### 3.4 Bandwidth of perceptual learning

To evaluate the transfer of perceptual learning at the cut-off spatial frequency to other frequencies, we compared the pre- and post-training spatial contrast sensitivity functions, applying the same method as in the previous study (Huang et al., 2008).

**Figure 6:**
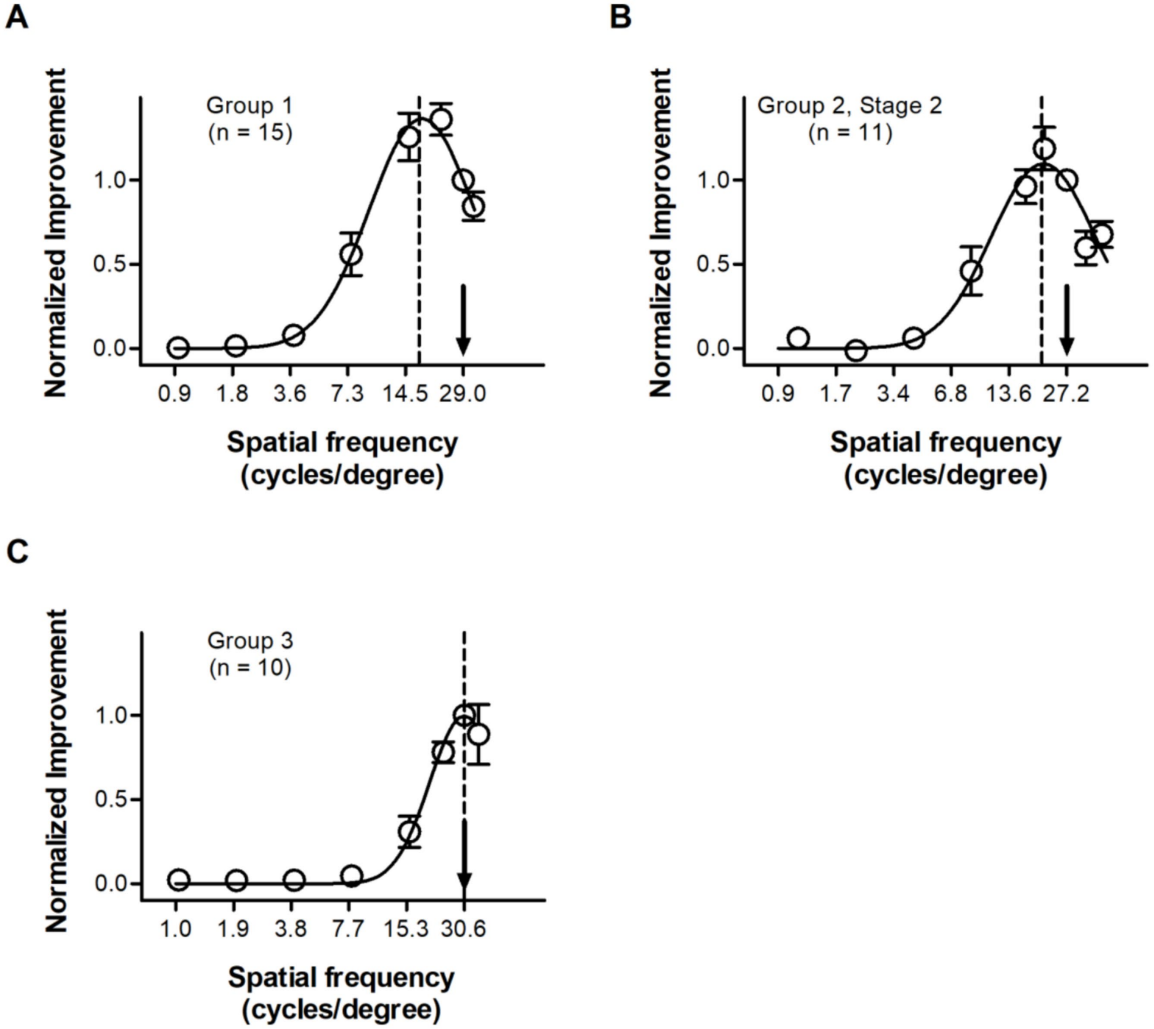
Average contrast sensitivity improvements as a Gaussian function of spatial frequency for Group 1 **(A)**, the second training stage of Group 2 **(B)** and Group 3 **(C)**. For each subject, the spatial frequencies and the improvements at these spatial frequencies were normalized to the training spatial frequency and the improvement at the training frequency, respectively. The Gaussian functions exhibited a peak shift for older adults (Group 1 and the second stage of Group 2), but not for young controls (Group 3). Arrows, average training spatial frequencies; dash lines, peak locations of the curves.

For Group 1, statistical analyses based on Gaussian model fits (Equations 4–7) and model comparisons (Equation 8 and 9) suggested that Equation 6 was the best one (for more details, see Table 2). The results of model fitting indicated a peak value about 1.37, a shift of the peak improvement about 0.72 octaves, and a bandwidth of perceptual learning (Equation 10) about 2.14 octaves, which can be seen in Figure 6A. Consistent with the results of model fitting, resampling analyses based on the best-fitting model confirmed that the bandwidth of perceptual learning was 2.18 ± 0.26 octaves, the perceptual learning at cut-off spatial frequencies resulted in larger improvements at lower spatial frequencies, with a statistically significant shift of peak improvement (0.68 ± 0.08 vs. 0, *p* < 0.001), and the improvements in the peak site were significantly larger than in the training sites (1.36 ± 0.08 vs. 1.00, *p* < 0.001). Note that the parameters averaged across calculations for each subject (peak value: 1.54 ± 0.10; peak shift: 0.65 ± 0.11; bandwidth: 1.97 ± 0.11) are statistically comparable from those calculated from average performances of all subjects described above (all *Ps* > 0.100).

**Table 2:** Parameters of four different Gaussian model fits and results of comparison.

Parameters	Group 1 (*n* = 15)	Group 2, Stage 2 (*n* = 11)	Group 3 (*n* = 10)
Model 1 (Equation 4)			
*Amp*	1.008	0.991	0.997
*σ*	2.136	1.574	0.846
*r* ^2^	0.964	0.944	0.999
Model 2 (Equation 5)			
*Amp*	1.037	0.958	0.974
*σ*	2.223	1.483	0.819
*baseline*	−0.029	0.003	0.024
*r* ^2^	0.964	0.945	0.999
Model 3 (Equation 6)			
*Amp*	1.366	1.098	0.997
*peak*	0.716	0.407	−0.012
*σ*	1.285	1.272	0.860
*r* ^2^	0.999	0.985	0.999
Model 4 (Equation 7)			
*Amp*	1.359	1.070	0.974
*peak*	0.712	0.399	−0.007
*σ*	1.268	1.293	0.827
*baseline*	0.010	0.003	0.024
*r* ^2^	0.999	0.985	0.999
Comparison between 1 & 2			
*F (1, 4)*	0.067	0.080	2.667
*p*	0.809	0.791	0.178
Comparison between 1 & 3			
*F* (1, 4)	117.000	10.555	0.000
*p*	<0.001	0.031	1.000
Comparison between 1 & 4			
*F* (2, 3)	48.000	4.295	3.750
*p*	0.005	0.132	0.148
Comparison between 3 & 4			
*F* (1, 3)	0.273	0.185	
*p*	0.638	0.696	
Best model	Model 3 (Equation 6)	Model 3 (Equation 6)	Model 1 (Equation 4)

For the second stage of Group 2, only the data of 11 subjects who showed statistically significant improvements at the training spatial frequency were used here (for more details, see Material and Methods). Consistent with the results of Group 1, statistical analyses suggested that Equation 6 was the best fitting model (for more details, see Table 2). The results of model fitting indicated a peak value about 1.10, a shift of the peak improvement about 0.41 octaves, and a bandwidth of perceptual learning about 2.12 octaves, as shown in Figure 6B. These results were confirmed by resampling analyses, which suggested a bandwidth of 2.02 ± 0.25 octaves, a peak value of 1.13 ± 0.08, and a statistically significant shift of peak improvement (0.42 ± 0.10 vs. 0, *p* < 0.001). Note that the parameters averaged across calculations for each subject (peak value: 1.23 ± 0.07; peak shift: 0.51 ± 0.09; bandwidth: 1.90 ± 0.13) are statistically comparable from those calculated from average performances of all subjects described above (all *Ps* > 0.100). The statistically significant shift of peak improvements found here was consistent with that found in Group 1, suggesting that the visual processing is less sensitive to training at the cut-off spatial frequency than at slightly lower frequencies in older adults.

For Group 3, statistical analyses suggested that the best fitting Gaussian models for young adults was Equation 4 (for more details, see Table 2). Note that only the data of 10 subjects who showed statistically significant improvements at the training spatial frequency were used here (for more details, see Material and Methods). It is clear that this model has no shift of the peak and is therefore very different from that of older adults (Equation 6). The model fitting of Equation 4 indicated a bandwidth of perceptual learning about 1.41 octaves and a peak value about 1.00, as shown in Figure 6C. These results were confirmed by resampling analyses. Which suggested a peak value of 0.97 ± 0.07 and a bandwidth of 1.47 ± 0.21 octaves. Note that the parameters calculated from those averaged across calculations for each subject (peak value: 1.00 ± 0.01; bandwidth: 1.50 ± 0.21) are statistically comparable from average performances of all subjects (peak value: *t*_9_ = 0.298, *p* = 0.773; bandwidth: *t*_9_ = 0.414, *p* = 0.689). Based on these results, it is clear that the bandwidth of perceptual learning at the cut-off spatial frequency was statistically comparable (*p* = 0.111) between young (1.50 ± 0.21 octaves) and older adults (1.90 ± 0.13, the second stage of Group 2), but the significant shift of the peak improvement was only found in older adults, not in young controls.

### 3.5 Recovery of age-related declines in SCSF

**Figure 7:**
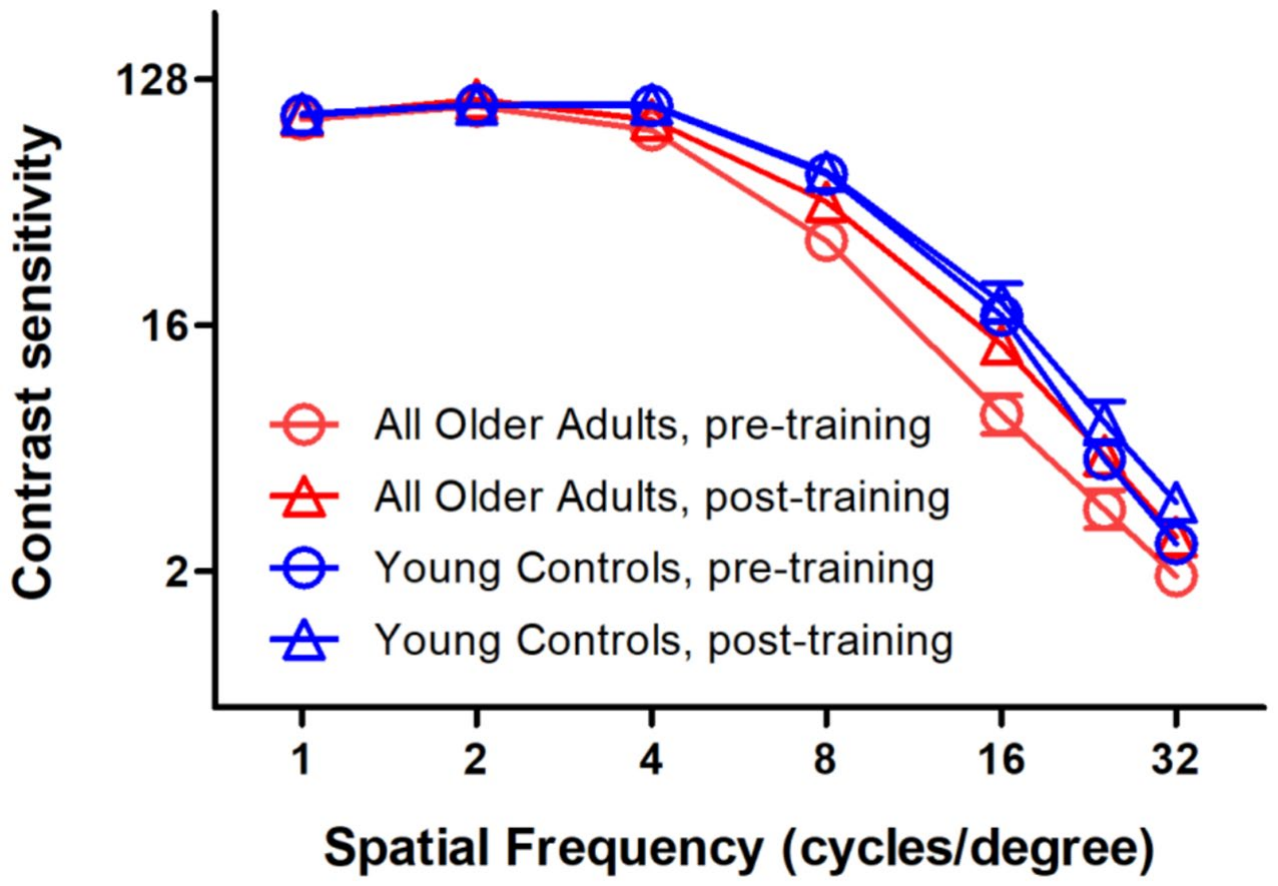
Comparisons of the spatial contrast sensitivity function between younger and older adults, both pre- and post-training. Despite demonstrating significantly lower SCSF than young controls at both time points, older adults reached post-training SCSF levels that were comparable to the pre-training performance of young adults. Red circles, pre-training SCSFs of all older adults; Red triangles, post-training SCSFs of all older adults; Blue circles, pre-training SCSFs of young controls; Red triangles, post-training SCSFs of young controls; error bars, between-subject SE.

When analyzing all older adults collectively, Figure 7 compares the SCSF between younger and older adults both pre- and post-training. Older adults demonstrated significantly lower SCSF than young controls at both time points (*p* < 0.001 for pre-training, and *p* = 0.014 for post-training). And the differences in SCSF were spatial frequency dependent (*p* < 0.001 for pre-training, and *p* = 0.001 for post-training), with more in spatial frequencies larger than 8 c/d. It should be noted that post-training SCSF in older adults reached levels statistically comparable to pre-training young adult performance (*p* = 0.725), strongly supporting the hypothesis that contrast perceptual learning is an efficient approach for the recovery of age-related declines in SCSF.

### 3.6 Improvements in visual acuity

**Figure 8:**
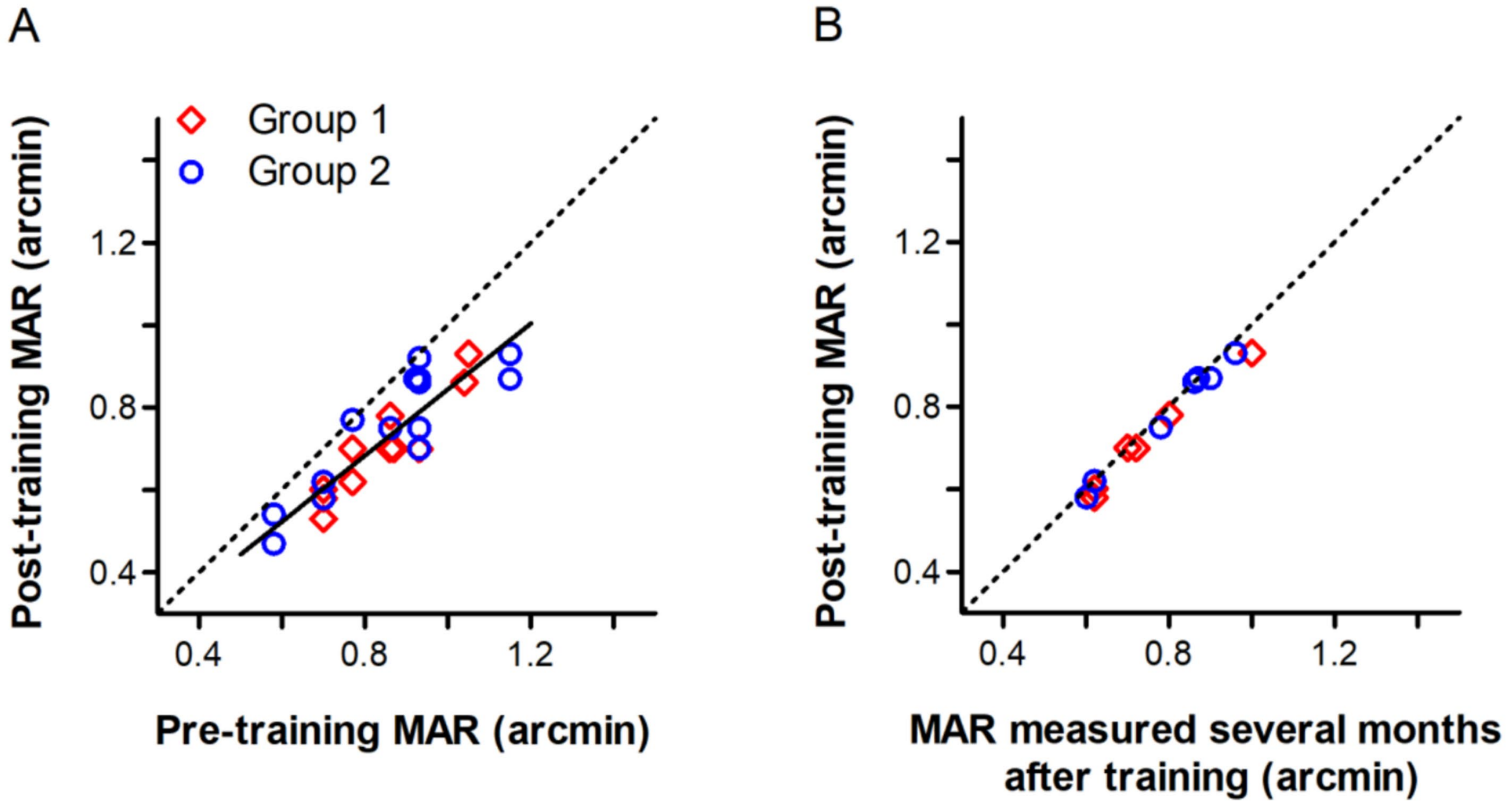
Visual acuity of older adults, expressed as minimum angel of resolution (MAR), measured pre-training, post-training and several (4–6) months after training. **(A)** A comparison of pre- and post-training visual acuity. The best fitting linear regression line (*r*^2^ = 0.803, *p* < 0.001) has a slope of 0.80, suggesting greater visual acuity improvements for subjects with initially worse visual acuities. **(B)** A comparison of post-training visual acuity and that measured several (4–6) months after training. The dashed line in both panel **(A,B)** is the identity line (slope = 1), indicating the prediction of no change.

As shown in Figure 8A, visual acuity of older adults in Group 1 and 2 measured with the Chinese Tumbling E Chart was also slightly improved after training. Taken all older subjects together, the best fitting linear regression line (*p* < 0.001) has a slope of 0.80, suggesting greater visual acuity improvements for subjects with initially worse visual acuities. These improvements could be retained for at least several months. Six subjects from Group 1 and seven subjects from Group 2 had their visual acuities retested 4–6 months after training, which were only slightly reduced (Figure 8B).

## 4 Discussion

In this study, we found contrast perceptual learning at the cut-off spatial frequency could improve SCSF and visual acuity in older adults. Especially, when compared to young controls, older adults showed a distinct transfer pattern of contrast perceptual learning at the cut-off frequency, characterized by an evident shift of the peak improvement. These findings provide new evidence that perceptual learning operates differently in younger and older adults. This advances our understanding of plasticity in the aging visual system and suggests that contrast perceptual learning could be an effective intervention for mitigating age-related declines in spatial contrast sensitivity.

As a fundamental visual function, spatial contrast sensitivity offers a more comprehensive measure of visual quality than visual acuity, and is widely used in both research and clinical practice (Ginsburg, 2003; Owsley, 2003). Numerous studies have investigated age-related declines in this function. In a large-sample study, Owsley et al. (1983) demonstrated that contrast sensitivity to low spatial frequencies remained stable throughout adulthood, whereas sensitivity to intermediate and high spatial frequencies declined significantly with age, as early as about forty years old. Similar patterns were also observed in both central and peripheral vision (Crassini et al., 1988), and across different luminance levels (Elliott et al., 1990). In this study, we also found significantly declined contrast sensitivity of older adults at intermediate and high spatial frequencies, consistent with the findings in previous studies.

As described in the Introduction, age-related declines in visual function are primarily driven from neural rather than ocular senescence. Despite this understanding, effective strategies for restoring the SCSF in older adults remain elusive. In a previous study, DeLoss et al. (2015) used a coarse orientation discrimination task for perceptual learning in older adults. They found that several days of training improved contrast sensitivity for Gabor patches in noisy conditions, but only minimal improvement was observed for noise-free stimuli at a relatively low spatial frequency (1.5 c/d). Critically, no significant enhancement was detected when measured with the Pelli-Robson Contrast Sensitivity Chart. In contrast, our study reveals that contrast perceptual learning at intermediate and high spatial frequencies induces substantial improvements in SCSF of older adults - precisely those ranges where previous studies have documented pronounced age-related declines in SCSF. Notably, training elevated older adults’ performance to levels comparable with pre-training young controls. Our results extend previous studies and suggest training can counteract age-related declines in spatial contrast sensitivity. Additionally, these findings, along with improvements in and retention of visual acuity in older adults, replicate earlier demonstrations that similar training protocols enhance SCSF in both healthy young adults (Zhou et al., 2012) and amblyopic patients (Zhou et al., 2006).

Since visual perceptual learning is recognized as reflecting the plasticity of the visual system (Dosher and Lu, 2017), the demonstration of training-induced improvements in older adults across multiple visual domains, including texture discrimination (Andersen et al., 2010), motion perception (Ball and Sekuler, 1986; Bower and Andersen, 2012; Bower et al., 2013), orientation discrimination (Deloss et al., 2014, 2015), letter and brightness discrimination (Ratcliff et al., 2006), global form perception (Mayhew and Kourtzi, 2013), contour integration (McKendrick and Battista, 2013), visual search (Rogers and Fisk, 1991), and the SCSF presented in this study, strongly suggests that the plasticity is well preserved in the aging visual system. This evidence naturally leads to a crucial subsequent question: To what extent does this preserved plasticity match that observed in normal young adults? Examination of the magnitude and specificity of perceptual learning may help address this question.

The magnitude and learning rates of perceptual learning between younger and older adults have been investigated in a number of studies, most of which reported comparable effects across age groups. For instance, Ball and Sekuler demonstrated that approximately 2,000 trials of training produced equivalent improvements in motion direction discrimination thresholds for both younger and older adults (Ball and Sekuler, 1986). Similarly, despite potential differences in underlying mechanisms, Bower and Andersen (2012) observed comparable training-induced enhancements in contrast sensitivity for moving sine-wave gratings and random-dot cinematograms (RDCs) between age groups (Bower and Andersen, 2012). Similar findings have been reported for orientation discrimination (Li et al., 2017), texture discrimination (Andersen et al., 2010; Chang et al., 2015), global visual form perception (Mayhew and Kourtzi, 2013) and contour integration (McKendrick and Battista, 2013), and visual sequence learning (Kruger et al., 2017). Notably, several studies have revealed more pronounced perceptual learning effects in older adults relative to their younger counterparts. Bower et al. (2013) found that older adults showed greater training-induced improvements in motion perception, assessed via duration thresholds in a coarse direction discrimination task, compared to young controls. Similarly, DeLoss et al. (2015) reported that older adults exhibited larger reductions in contrast thresholds for orientation discrimination in noisy conditions after several days of training. More strikingly, Astle et al. (2015) observed that older adults achieved faster learning rates in a peripheral word identification task, ultimately reaching performance levels equivalent to those of post-training younger participants. Consistent with these findings, our study demonstrated significantly greater improvements in not only the contrast sensitivity at cut-off spatial frequencies but also the whole SCSF among older adults, elevating their post-training performance to levels comparable with young controls before training. These results suggest that the aging visual system retains plasticity comparable to or even exceeding that of young individuals, particularly in contrast sensitivity processing.

It is important to note that the magnitude of perceptual learning in older adults can be influenced by some factors, such as training duration and intensity. In the present study, older adults in Group 2 showed limited improvements in SCSF during the first stage of training, where low training intensity (about 143 trials/spatial frequency) was used, with performance plateauing by the end of this stage. However, when the training intensity increased to 1,000 trials/spatial frequency in the second stage, further improvements were observed. These findings demonstrate a clear relationship between the magnitude of perceptual learning and the training intensity in older adults, similar to those found in young adults (Aberg et al., 2009; Hussain et al., 2009). In addition, although all subjects completed their training with the same criterion in the present study, older adults required slightly more training sessions to reach the plateau performance compared to young adults. Consequently, while learning rates were similar during the initial days of training, as reported in several previous studies, it is also possible that the ultimate magnitude of perceptual learning could be greater in older than in younger adults. Since few studies have systematically explored these issues, future research should investigate the effects of varying training duration and intensity across multiple visual tasks to further elucidate age-related differences in the magnitude of perceptual learning.

Specificity serves as a hallmark characteristic of visual perceptual learning, and interpreting it within the framework of brain networks helps locate the site of plasticity (Dosher and Lu, 2017; Lu and Dosher, 2022). Therefore, age-related differences in the specificity of visual perceptual learning may reflect changes in plasticity within the aging visual cortex. Several studies have explored this issue. For example, in a texture discrimination task, Andersen et al. (2010) found that the learning effects were specific to the stimulus-presented quadrant for both younger and older adults, indicating preserved spatial location specificity across age groups. Similarly, Bower and Andersen observed partial transfer of motion perception learning between moving gratings and RDCs in both younger and older observers, suggesting comparable transfer patterns (Bower and Andersen, 2012). DeLoss et al. (2015) reported analogous findings for contrast perceptual learning in an orientation discrimination task. Critically, these studies consistently demonstrated similar specificity of visual perceptual learning in younger and older adults. In contrast, out study examined transfer effects of contrast perceptual learning from the trained spatial frequency to adjacent frequencies and observed a distinct shift in the peak improvement of the transfer Gaussian function for older adults, which was absent in young controls. Additionally, the bandwidth of the transfer function was broader for older adults, albeit only marginally significant statistically. These findings indicate an age-related alteration in specificity. Nevertheless, our results do not necessarily contradict earlier work. Visual perceptual learning has been suggested to involve multiple representations, including low-level representation of a single feature (for example, spatial frequency in the present study), mid-level representation of patterns composed of several simple features (for example, texture pattern in the study of Andersen et al., and RDCs in the study of Bower and Andersen), and high-level representation of objects and natural scenes, of trained stimuli (Lu and Dosher, 2022). Therefore, it is possible that the neural networks underlying various tasks exhibit different characteristics, such as specificity, during normal aging. Collectively, all these findings suggest that age-related changes in visual plasticity are more complex than previously appreciated and may involve diverse neural mechanisms.

Previous studies have investigated the mechanisms underlying visual perceptual learning in texture discrimination tasks among older adults, with particular attention to age-related differences compared to younger populations. By using fMRI, Chang et al. (2015) compared the area size of V1, V2, and V3, defined by retinotopic mapping, in younger and older adults, and investigated the association between these morphologic measures and the magnitude of perceptual learning in the texture discrimination task. Their results revealed significant age-related reductions in area size of all three regions, with the most pronounced reduction in V1 and the least in V3. Notably, the magnitude of perceptual learning in older adults correlated specifically with V3 size but showed no association with any visual areas in young controls. Complementing these findings, Yotsumoto et al. (2014) reported training-induced increases in fractional anisotropy in white matter beneath early visual cortex, particularly V3, in older adults, suggesting that visual perceptual learning of older individuals involves reorganization of white matter. In contrast, no significant changes in fractional anisotropy were observed for young controls. Collectively, these results suggest a marked age-related reduction in V1 plasticity, with perceptual learning gains in older adults predominantly mediated by compensatory plasticity in other area, such as V3, in the early visual cortex. However, this compensatory mechanism does not seem adequate to explain the findings of the present study. In contrast, our results support the hypothesis that there is substantial residual plasticity of V1 in the aging visual system. Although direct evidence remains limited, several observations support our hypothesis.

Firstly, the primary visual cortex is traditional regarded as the neural substrate for spatial vision, particularly for visual perception at high spatial frequencies. Neurons in the primate visual cortex demonstrate characteristic tuning to spatial frequency (Schiller et al., 1976c), orientation (Schiller et al., 1976b), and moving direction (Schiller et al., 1976a), with their firing rates systematically modulated by stimulus contrast. Electrophysiological and behavior studies have demonstrated this functional specialization across species. In rhesus monkeys, the optimal spatial frequencies of V1 neurons at retinal eccentricities of 2–5 degrees reach a high level of approximately 8.0 c/d (Foster et al., 1985), much higher than those (about 3.0 c/d) observed in cat area 17 neurons (Movshon et al., 1978). Consistently, the spatial resolution investigated in behavior examinations exceeds 30 c/d in the central visual field of monkeys (Merigan and Katz, 1990), about 3–4 times greater than that (8–9 c/d) of cats (Jacobson et al., 1976; Hall and Mitchell, 1991). Notably, this selectivity for high spatial frequencies appears most pronounced in V1 compared to other early visual areas. The optimal spatial frequencies of neurons in other area of the early visual cortex, such as V2 in monkeys (Foster et al., 1985) and area 18 (Movshon et al., 1978) in cats, are much lower than those in the primary visual cortex. Based on this hierarchical organization, the observed improvements at the cut-off spatial frequencies would be expected to primarily reflect V1 plasticity, rather than changes in other early visual areas.

Secondly, neurons in the primary visual cortex exhibit age-related functional declines, as extensively documented in previous studies. Electrophysiological recordings in primates and cats demonstrated that aging visual cortical neurons display elevated spontaneous activity, increased visually evoked responses, and reduced signal-to-noise ratios (Schmolesky et al., 2000; Hua et al., 2006). Notably, both orientation and direction selectivity were found to be significantly attenuated in aged rhesus monkeys (Schmolesky et al., 2000; Fu et al., 2012) and cats (Hua et al., 2006) compared to their younger counterparts. Similar age-dependent deterioration was also observed in contrast response functions (Yang et al., 2008). Crucially, V1 neurons in aged monkeys exhibited reduced optimal spatial frequencies and lower spatial resolution relative to young adults (Zhang et al., 2008), which provides a mechanistic framework for understanding our behavioral observations. The downward shift in neuronal optimal spatial frequencies may explain the reduced contrast sensitivity for high spatial frequencies observed in older adults. Notably, the paucity of neurons selective for cut-off spatial frequency due to age-related frequency degradation may force the system to compensate by relying more heavily on neurons with optimal frequencies just below the cut-off, leading to the age-associated shift in peak improvement and slightly broader bandwidth of the Gaussian transfer function observed in the present study. Collectively, the concordance between these neural properties and our behavioral demonstration of training-induced enhancement in older adults is consistent with the hypothesis that the observed perceptual improvements reflect functional plasticity within the primary visual cortex.

Finally, electrophysiological studies have demonstrated that contrast perceptual learning induces modifications in neuronal response properties within the primary visual cortex, including refined orientation tuning, shifts in optimal spatial frequency tuning and changes in contrast response function. For instance, training with a fine orientation discrimination task led to sharpening of orientation tuning of V1 neurons in rhesus monkeys (Schoups et al., 2001). After training with a low-spatial-frequency grating orientation identification task, neurons in area 17 (V1) that preferentially responded to stimuli presented to the trained eye exhibited significantly higher contrast sensitivity compared to those driven by the untrained eye (Hua et al., 2010). Notably, this effect was specific to the trained spatial frequency. A subsequent study (Ren et al., 2016) employed the same orientation identification task but used gratings at the cut-off spatial frequency (the same as in the present study). Following training, cats showed behavioral improvements in grating acuity. At the neuronal level, V1 population exhibited a shift in optimal spatial frequency toward higher frequencies. Crucially, these neuronal changes in optimal spatial frequency were significantly correlated with the observed behavioral improvements in acuity among training cats. These findings suggest that striate cortex neurons may mediate training-induced perceptual enhancements for high-spatial-frequency stimuli, supporting our hypothesis previously described.

Notably, while our findings support the hypothesis that substantial plasticity within the primary visual cortex underlies the training effects observed in older adults, alternative explanations remain plausible. Based on the reweighting theory (Dosher and Lu, 1998; Dosher and Lu, 2017; Lu and Dosher, 2022), higher-order visual cortex may also be involved in the perceptual learning of older adults. It has been suggested that visual perceptual learning involves two mechanisms, representation enhancement and information reweighting. The former may stem from altered responses or tuning properties of neurons in early visual cortical areas, as we discussed above, whereas the latter involves up-weighting relevant and down-weighting irrelevant sensory signals during decision-making. Consequently, information reweighting enhances behavioral performance by improving the read-out of task-relevant information, modulating lateral interactions, and/or incorporating top-down feedback. Additionally, training may lead to task familiarity and improved attentional engagement in older adults when performing the perceptual task, which in turn contributes to enhanced performance.

Before concluding, we must address the potential limitations. The present study is limited to behavioral data and lacks direct neurobiological validation. Consequently, the functional significance of our key findings that older adults showed broader bandwidths and shifted peaks in their transfer functions remains unclear. It is uncertain whether the observed low-frequency shift reflects changes in cortical tuning, a compensatory reliance on alternative neural populations, or purely behavioral strategies. Future studies are needed to resolve this question.

In conclusion, we demonstrated that contrast perceptual learning can substantially improve the spatial contrast sensitivity and visual acuity in older adults. Compared to young controls, older adults exhibited more training-induced improvements in SCSF, along with a distinct low-frequency shift in peak improvement and a slightly boarder bandwidth of the Gaussian transfer function. These findings indicate that neural plasticity is well preserved in the aging visual system, suggesting that perceptual learning could serve as an effective intervention for ameliorating age-related visual decline.

## Data Availability

The original contributions presented in the study are included in the article/supplementary material, further inquiries can be directed to the corresponding author.
